# MicroRNA and mRNA expression profiling in rat acute respiratory distress syndrome

**DOI:** 10.1186/1755-8794-7-46

**Published:** 2014-07-28

**Authors:** Chaoqun Huang, Xiao Xiao, Narendranath Reddy Chintagari, Melanie Breshears, Yang Wang, Lin Liu

**Affiliations:** 1 Department of Physiological Sciences, Lundberg-Kienlen Lung Biology and Toxicology Laboratory, Stillwater, OK, USA; 2 Department of Pathobiology, Oklahoma State University, Stillwater, OK, USA; 3 Oklahoma Center for Respiratory and Infectious Diseases, Oklahoma State University, Stillwater, OK, USA; 4 Department of Physiological Sciences, Oklahoma State University, 264 McElroy Hall, Stillwater, OK 74078, USA

**Keywords:** MicroRNA, mRNA, Microarray, ARDS

## Abstract

**Background:**

Acute respiratory distress syndrome (ARDS) is characterized by pulmonary epithelial injury and extensive inflammation of the pulmonary parenchyma. Systematic analyses of microRNA (miRNA) and mRNA expression profiling in ARDS provide insights into understanding of molecular mechanisms of the pathogenesis of ARDS. The objective of this study was to identify miRNA and mRNA interactions in a rat model of ARDS by combining miRNA and mRNA microarray analyses.

**Methods:**

Rat model of ARDS was induced by saline lavage and mechanical ventilation. The expression profiles of both mRNAs and miRNAs in rat ARDS model were performed by microarray analyses. Microarray data were further verified by quantitative RT-PCR. Functional annotation on dys-regulated mRNAs and miRNAs was carried out by bioinformatics analysis.

**Results:**

The expression of 27 miRNAs and 37 mRNAs were found to be significantly changed. The selected miRNAs and genes were further verified by quantitative real-time PCR. The down-regulated miRNAs included miR-24, miR-26a, miR-126, and Let-7a, b, c, f. The up-regulated miRNAs were composed of miR-344, miR-346, miR-99a, miR-127, miR-128b, miR-135b, and miR-30a/b. Gene ontology and functional annotation analyses indicated that up-regulated mRNAs, such as Apc, Timp1, and Sod2, were involved in the regulation of apoptosis. Bioinformatics analysis showed the inverse correlation of altered miRNAs with the expression of their predicted target mRNAs. While Sod2 was inversely correlated with Let-7a, b, c, f., Ebf1 and Apc were inversely correlated with miR-24 and miR-26a, respectively. miR-26a, miR-346, miR-135b, miR-30a/b, miR-344, and miR-18a targeted multiple altered mRNAs. Gabrb1, Sod2, Eif2ak1, Fbln5, and Tspan8 were targeted by multiple altered miRNAs.

**Conclusion:**

The expressions of miRNAs and mRNAs were altered in a rat model of ARDS. The identified miRNA-mRNA pairs may play critical roles in the pathogenesis of ARDS.

## Background

Acute Respiratory Distress Syndrome (ARDS) is a severe lung disease that leads to a low oxygen level in the blood [1]. ARDS usually occurs in sepsis [2] or with other major injuries that may lead to multiple organ failure [3]. Lung inflammation, hypoxemia and non-cardiogenic pulmonary edema formation are characteristic features of ARDS [4]. Approximately 200,000 ALI/ARDS cases per year are found in the U.S. and a mortality is as high as 40% [5].

The main sites of cell injury in ARDS are vascular endothelium and alveolar epithelium. Neutrophils contribute to lung inflammation and play important roles in the pathogenesis and progression of ARDS. Lung injures cause the activation and migration of neutrophils into the pulmonary interstitium and alveolar space. The activated neutrophils damage endothelial and epithelial cells [6]. Endothelial injury leads to the increases in capillary permeability and effusion of protein-rich fluid into alveolar airspace [7]. Damage to alveolar epithelial cells causes increased entry of fluid into the alveolar lumens, decreased clearance of fluid from the alveolar airspace, and decreased production of surfactant [8].

MicroRNAs (miRNAs) are a class of non-coding small RNAs with approximately 22 nucleotides in length. They are important regulators of post-transcriptional gene expression. The mature miRNAs control gene expression by binding the 3'-untranslated region (3'-UTR) of its target gene, resulting in either reduced protein translation or degradation of mRNA. Many miRNAs are expressed in the lung [9,10]. miR-17, miR-92a and miR-127 have been shown to regulate lung development [11,12]. VEGF is a well-defined ARDS-associated candidate gene, and is a target of miR-126 [13,14]. The miRNA profiling was used to identify the miRNAs involved in the pathogenesis of various lung diseases such as ventilator-induced lung injury [15], bronchopulmonary dysplasia (BPD) [16,17], chronic obstructive pulmonary disease (COPD) [18,19], and idiopathic pulmonary fibrosis (IPF) [20,21]. However, it remains to be investigated whether miRNAs are involved in the pathogenesis of ARDS.

Genetic and environmental factors influence the susceptibility and the severity of ARDS [7]. For example, individuals with similar environmental factor exposure and prior diseases differ in their risk of developing ARDS or in their survival following ARDS, indicating a role of genetic component in the disease outcome [22,23]. Thus, it is important to investigate the contribution of genetic factors to ARDS including gene-gene and miRNA-gene interactions [24]. Because of complex and heterogenous mechanisms of human ARDS, we used a rat model of ARDS induced by saline lavage and mechanical ventilation to perform miRNA and mRNA microarray analyses simultaneously, aiming to identify miRNA-mRNA interactions and to understand the impact of these interactions on the pathogenesis of ARDS.

## Results

### ARDS model

**Figure 1:**
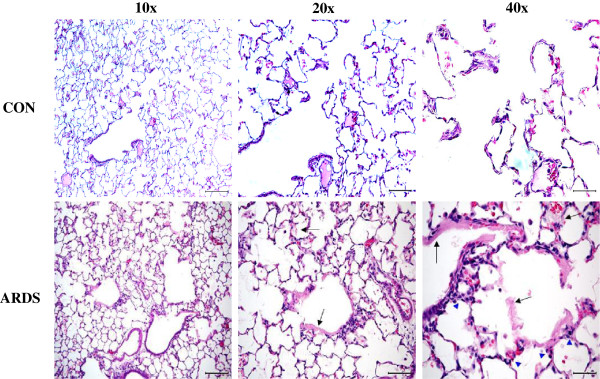
**ARDS-induced histopathological changes in rat lungs.** Rats were subjected to surfactant depletion by repeated lung lavages (10 times) with saline to experimentally induce ARDS. The controls were non-lavaged and non-ventilated rats, maintained at room air until the collection of lung tissue. Shown are representative images. Arrows indicate hyaline membranes. Scale bars: 10×, 200 μm; 20×, 100 μm; and 40×, 50 μm.

Repeated lavage to deplete lung surfactant, followed by mechanical ventilation has been used as an experimental model of ARDS in rats [25]. Histopathological examination of lung specimens confirmed the presence of mild to moderate pulmonary lesions characterized by extensive interstitial edema, and neutrophilic infiltration in the alveolar septa and lumens in this model of ARDS (Figure 1 and Table 1). Based on the scoring system (see the Methods), all of the parameters for controls are 0 and thus were not included in Table 1. There was minimal evidence of septal necrosis and moderate hyaline membrane formation within alveolar lumens.

**Table 1:** Histopathological scores of rat ARDS (n = 19 rats)

**Histopathological lesion**		**ARDS**	
		**Median (Range)**	**Mean ± SEM**
Interstitial	Neutrophils	2.00 (1;3)	2.11 ± 0.19
	Edema	2.50 (1;3)	2.42 ± 0.19
Intraalveolar	Neutrophils	1.56 (1;3)	1.52 ± 0.18
	Edema	1.39 (1;3)	1.60 ± 0.18
Alveolar septal necrosis		1.00 (1;3)	1.42 ± 0.23
Hyaline membranes		1.89 (1;3)	1.51 ± 0.20

### miRNA and mRNA expression profiles of rat ARDS

**Figure 2:**
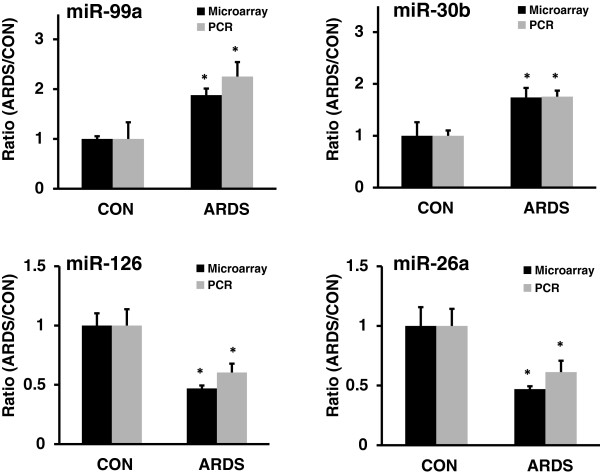
**Validation of miRNA microarray data by real-time PCR.** Small RNA was extracted from control (CON) and ARDS rat lungs. The expression of miRNAs relative to U6 RNA was determined by real-time PCR. The results were expressed as a ratio of ARDS to CON. Data are presented as means ± S.D. from 4 animals, each assay performed in duplicate. *p < 0.05, v.s. CON. Microarray: SAM test; Real-time PCR: *t*-test.

To identify the altered miRNAs in the rat lung of ARDS, we performed miRNA profiling using an in-house printed microarray containing 227 rat miRNAs. The miRNA microarray data were deposited to the GEO database (http://www.ncbi.nlm.nih.gov/geo/, GSE57223). The results in Table 2 showed that the expression of 27 miRNAs was significantly changed based on SAM test (q < 0.05). Among them, 20 miRNAs were up-regulated and 7 miRNAs were down-regulated. The down-regulated miRNAs included miR-24, miR-26a, miR-126, and Let-7 family members. The up-regulated miRNAs included miR-99a, miR-127, miR-128b, miR-135b, miR-30a, and miR-30b. Several selected miRNAs were validated using real-time PCR. miR-99a and miR-30b were confirmed to be the up-regulated miRNAs in ARDS, while miR-126 and miR-26a were confirmed to be down-regulated miRNAs in ARDS (Figure 2).

**Table 2:** Altered miRNAs in rat ARDS

**miRNA**	**Fold change**	**q-value**
rno-miR-346	2.44	<0.05
rno-miR-341	2.31	<0.05
rno-miR-344	2.09	<0.05
rno-miR-135b	2.04	<0.05
rno-miR-99a	1.88	<0.05
rno-miR-349	1.83	<0.05
mmu-miR-380-5p	1.82	<0.05
rno-miR-19a	1.76	<0.05
rno-miR-128b	1.75	<0.05
rno-miR-30b	1.74	<0.05
rno-Let-7d*	1.69	<0.05
rno-miR-30a-3p	1.66	<0.05
rno-miR-18	1.65	<0.05
rno-miR-210	1.64	<0.05
rno-miR-127	1.61	<0.05
rno-miR-333	1.59	<0.05
rno-miR-207	1.59	<0.05
rno-miR-129	1.55	<0.05
rno-miR-337	1.51	<0.05
rno-miR-215	1.51	<0.05
rno-Let-7f	0.59	<0.05
rno-miR-24	0.58	<0.05
rno-Let-7a	0.52	<0.05
rno-Let-7b	0.5	<0.05
rno-Let-7c	0.48	<0.05
rno-miR-126	0.47	<0.05
rno-miR-26a	0.47	<0.05

A list of increased and decreased miRNAs in rat ARDS (n = 4 animals) compared to normal lung samples with a q value of < 0.05 (SAM) and a fold change of ≥1.5.

**Figure 3:**
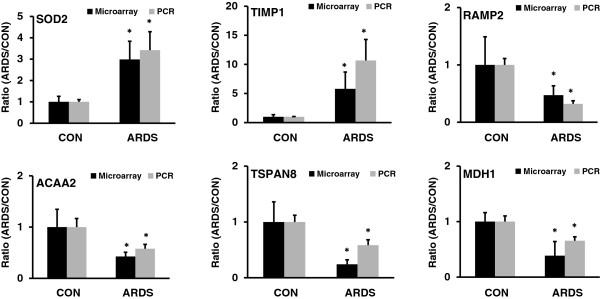
**Validation of mRNA microarray data by real-time PCR.** Total RNA was extracted from control (CON) and ARDS rat lungs. The expression of mRNAs relative to 18S rRNA was determined by real-time PCR. The results were expressed as a ratio of ARDS to CON. Data are presented as means ± SD from 4 animals, each assay performed in duplicate. *p < 0.05, v.s. CON. Microarray: SAM test; Real time PCR: *t*-test.

DNA microarray was performed to identify the altered mRNAs in ARDS using an in-house printed DNA microarray containing 10,000 rat genes. The microarray data were deposited to the GEO database: http://www.ncbi.nlm.nih.gov/geo/, GSE57011. The expression of 37 genes was significantly changed based on a q value of <0.05 (SAM test) and a fold change of ≥ 2 (Table 3). Among them, eleven genes were up-regulated and twenty six genes were down-regulated. Sod2 (Superoxide dismutase 2) and Timp1 (Metalloproteinase inhibitor 1) modulate lung injury [26,27]. Ramp2 [Receptor (calcitonin) activity modifying protein 2], Acaa2 (Acetyl-Coenzyme A acyltransferase 2), Mdh1 (Malate dehydrogenase 1, NAD), and Tspan8 (Tetraspanin 8) are enriched mRNAs in the lungs and are involved in lung disease [28,29]. These mRNAs were selected for validation by qRT-PCR. The results in Figure 3 showed that Sod2 and Timp1 were confirmed to be up-regulated in ARDS. Ramp2, Acaa2, Mdh1, and Tspan8 were confirmed to be down-regulated in ARDS.

**Table 3:** Changed mRNAs in rat ARDS

**Gene**	**Full name**	**Fold change**	**q-value**
Mt3	Metallothionein 3	43.49	<0.05
S100a9	S100 calcium binding protein A9 (calgranulin B)	6.81	<0.05
Prdx6	Peroxiredoxin-6	5.89	<0.05
TIMP1	Metalloproteinase inhibitor 1	5.79	<0.05
Ccl2	Chemokine (C-C motif) ligand 2	3.90	<0.05
Sod2	Superoxide dismutase 2, mitochondrial	2.98	<0.05
Lcn2	Lipocalin 2	2.59	<0.05
Ifrd1	Interferon-related developmental regulator 1	2.16	<0.05
Apc	Adenomatosis polyposis coli	2.02	<0.05
Ebf1	Early B-cell factor 1	2.00	<0.05
Mt2a	metallothionein-2 and metallothionein-1 genes	2.00	<0.05
Cyb5	Cytochrome b5	0.52	<0.05
L20990	T cell receptor	0.51	<0.05
Serpinh1	Serine (or cysteine) proteinase inhibitor, clade H, member 1	0.5	<0.05
S100a4	S100 calcium-binding protein A4	0.49	<0.05
Eif2ak1	Eukaryotic translation initiation factor 2-alpha kinase 1	0.49	<0.05
Fbln5	Fibulin 5	0.48	<0.05
Aco2	Aconitase 2, mitochondrial	0.48	<0.05
Akr1b4	Aldo-keto reductase family 1, member B4 (aldose reductase)	0.47	<0.05
Lct	Lactase-phlorizinhydrolaseLactasePhlorizin hydrolase	0.47	<0.05
Slc25a11	Solute carrier family 25 (mitochondrial carrier; oxoglutarate carrier), Member 11	0.47	<0.05
Ramp2	Receptor (calcitonin) activity modifying protein 2	0.47	<0.05
G8	G8 gene	0.46	<0.05
Acaa2	Acetyl-Coenzyme A acyltransferase 2 (mitochondrial 3-oxoacyl-Coenzyme A thiolase)	0.43	<0.05
Gabrb1	Gamma-aminobutyric acid (GABA-A) receptor, subunit beta 1	0.43	<0.05
M13801	Ig germline alpha H-chain C-region gene	0.42	<0.05
Septin 5	Septin 5	0.4	<0.05
Mdh1	Malate dehydrogenase 1, NAD (soluble)	0.39	<0.05
Psma4	Proteasome (prosome, macropain) subunit, alpha type 4	0.39	<0.05
Alad	Aminolevulinate, delta-, dehydratase	0.38	<0.05
Igfbp6	Insulin-like growth factor binding protein 6	0.37	<0.05
Ces3	Carboxylesterase 3	0.35	<0.05
U06230	protein S mRNA	0.31	<0.05
Fgfr4	Fibroblast growth factor receptor subtype 4 (FGFR4) mRNA	0.27	<0.05
Lim2	Lens intrinsic membrane protein 2	0.24	<0.05
Tspan8	Tetraspanin 8	0.24	<0.05
Gnrh1	Progonadoliberin-1Gonadoliberin-1Prolactin release-inhibiting factor 1	0.08	<0.05

A list of increased and decreased mRNAs in rat ARDS (n = 4 animals) when compared to normal lung samples (n = 4 animals) with a q value of < 0.05 (SAM) and a fold change of ≥ 2.

### Functional annotation of the identified genes

Functional annotation of the identified genes was carried out using David gene-GO term enrichment analysis and functional annotation clustering. The DAVID functional annotation clustering uses an algorithm to explore relationships among the annotation terms via the degrees of co-associated genes. The similar, redundant, and heterogeneous annotation contents from the same or different resources were clustered into annotation groups due to their similar biological meaning. We used DAVID default population (Rattus norvegicus) background in enrichment calculation. The functional annotation clustering was done with default parameters. Classification stringency was set as medium. The raw p values were used in functional annotation. The results in Table 4 showed that the up-regulated genes were involved in two functional clusters with an enrichment score of over 1.3. The results in Table 5 showed the down-regulated genes were involved in two functional clusters with an enrichment score of over 1.3. A more detailed list of genes was provided in Additional file 1.

**Table 4:** Functional annotation clustering of up-regulated genes

**Annotation cluster 1**	**Enrichment score: 2.44**			
**Category**	**Term**	**Count**	**%**	**P value**
GOTERM_BP_FAT	GO:0019725 ~ cellular homeostasis	5	56	< 0.001
GOTERM_BP_FAT	GO:0055066 ~ di-, tri-valent inorganic cation homeostasis	3	33	0.003
GOTERM_BP_FAT	GO:0055080 ~ cation homeostasis	3	33	0.005
GOTERM_BP_FAT	GO:0006873 ~ cellular ion homeostasis	3	33	0.010
GOTERM_BP_FAT	GO:0055082 ~ cellular chemical homeostasis	3	33	0.010
GOTERM_BP_FAT	GO:0050801 ~ ion homeostasis	3	33	0.012
GOTERM_BP_FAT	GO:0048878 ~ chemical homeostasis	3	33	0.018
**Annotation cluster 2**	**Enrichment score: 1.82**			
GOTERM_BP_FAT	GO:0043066 ~ negative regulation of apoptosis	3	33	0.008
GOTERM_BP_FAT	GO:0043069 ~ negative regulation of programmed cell death	3	33	0.008
GOTERM_BP_FAT	GO:0060548 ~ negative regulation of cell death	3	33	0.008
GOTERM_BP_FAT	GO:0030097 ~ hemopoiesis	3	33	0.009
GOTERM_BP_FAT	GO:0048534 ~ hemopoietic or lymphoid organ development	3	33	0.011
GOTERM_BP_FAT	GO:0002520 ~ immune system development	3	33	0.012
GOTERM_BP_FAT	GO:0042981 ~ regulation of apoptosis	3	33	0.039
GOTERM_BP_FAT	GO:0043067 ~ regulation of programmed cell death	3	33	0.040
GOTERM_BP_FAT	GO:0010941 ~ regulation of cell death	3	33	0.041

The functional annotation of mRNA expression profile was conducted by DAVID software (http://david.abcc.ncifcrf.gov) [30,31]. Annotation Cluster: a group of terms having similar biological functions. Enrichment Score: The geometric mean (in -log scale) of member's p-values in a corresponding annotation cluster is the rank of their biological significance. The higher an enrichment score, the more enriched genes in that group. An enrichment score of >1.3 is used for a cluster to be statistically significant. P-Value: The p-values associated with each annotation terms are the Fisher Exact Score shown in the regular chart report for the same terms. Count: Genes involved in the terms. %: Percentage of involved genes over total up-regulated genes correlated to altered miRNAs.

**Table 5:** Functional annotation clustering of down-regulated genes

**Annotation cluster 1**	**Enrichment score: 1.38**			
**Category**	**Term**	**Count**	**%**	**P value**
SP_PIR_KEYWORDS	Disulfide bond	8	38	0.014
SP_PIR_KEYWORDS	Glycoprotein	9	43	0.034
UP_SEQ_FEATURE	Signal peptide	8	38	0.035
SP_PIR_KEYWORDS	Signal	8	38	0.037
UP_SEQ_FEATURE	Glycosylationsite: N-linked (GlcNAc…)	8	38	0.072
UP_SEQ_FEATURE	Disulfide bond	6	29	0.117

The functional annotation of mRNA expression profile was conducted by DAVID software (http://david.abcc.ncifcrf.gov) [30,31]. Annotation Cluster: A group of terms having similar biological functions. Enrichment Score: The geometric mean (in -log scale) of member's p-values in a corresponding annotation cluster represents the rank of their biological significance. An enrichment score of >1.3 is used for a cluster to be statistically significant. The higher an enrichment score, the more enriched genes in that group. P-Value: The p-values associated with each annotation terms are the Fisher Exact Score shown in the regular chart report for the same terms. Count: Genes involved in the terms. %: Percentage of involved genes over total down-regulated genes correlated to altered miRNAs.

More than 33% of the up-regulated genes were involved in biological processes such as cellular homeostasis and regulation of apoptosis. The genes involved in apoptosis were Apc (Adenomatosis polyposis coli), Timp1, and Sod2. Interestingly, a large amount of down-regulated and up-regulated genes encoded the proteins that were modified by a disulfide bond and glycosylation. Moreover, the down-regulated genes were enriched in the functional groups of acetylation and ion binding.

**Figure 4:**
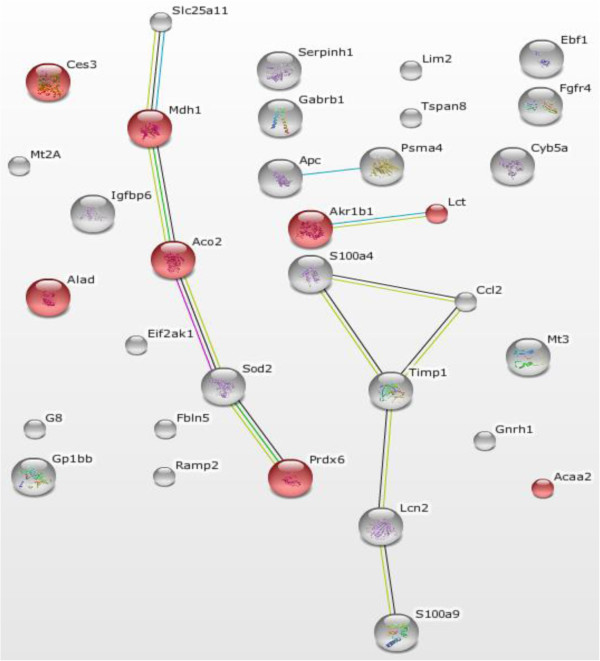
**STRING analysis of pathway enrichment and interaction in the altered mRNA in rat ARDS.** Eight mRNAs, Lct, Mdh1, Akr1b1, Alad, Ces3, Aco2, Prdx6, and Acaa2 were involved in the metabolic pathways (pink color). Eleven interactions were observed in the protein products of altered mRNAs (connected node).

STRING is a web-based tool to explore GO annotation, protein-protein interactions and KEGG pathway. STRING GO enrichment is typically GO analysis including 3 annotations-biological process, cell compartment, and molecular function. Table 6 listed GO enrichments of the down-regulated genes in ARDS as identified by STRING GO enrichment analysis. No GO enrichment was identified for the up-regulated genes in ARDS. Using STRING, we also performed KEGG pathway enrichment analysis of the altered mRNAs in ARDS (Table 7). Eight genes including Lct (lactase-phlorizin hydrolase), Mdh1 (alate dehydrogenase 1, NAD), Akr1b1(aldo-keto reductase family 1, member B1), Alad (delta-aminolevulinic acid dehydratase), Ces3 (carboxylesterase 3), Aco2 (aconitate hydratase, mitochondrial precursor), Prdx6 (peroxiredoxin-6), and Acaa2 (acetyl-coenzyme A acyltransferase 2) were involved in the metabolic pathways. STRING analysis of protein-protein interactions revealed 11 interactions of the protein products of altered mRNAs (Figure 4).

**Table 6:** GO enrichments of the down-regulated genes in ARDS using STRING analysis

**Go enrichment**	**GO_id**	**Term**	**Number of genes**	**p-value**	**Involved genes**
Biological process	GO:0046501	Protoporphyrinogen IX metabolic process	2	1.15E-04	Eif2ak1, Alad
	GO:0030198	Extracellular matrix organization	2	4.84E-03	Ramp2, Flbn5
	GO:0043062	Extracellular structure organization	2	4.84E-03	Ramp2, Flbn5
	GO:0044242	Cellular lipid catabolic process	2	5.73E-03	Acaa2, Ces3
	GO:0046777	Protein autophosphorylation	2	8.67E-03	Eif2ak1, Fgfr4
	GO:0030162	Regulation of proteolysis	2	1.25E-02	Serpinh1, Fgfr4
	GO:0055114	Oxidation-reduction process	3	1.33E-02	Acaa2, Cyb5, Mdh1
	GO:0070613	Regulation of protein processing	2	1.70E-02	Serpinh1, Fgfr4
	GO:0045471	Response to ethanol	2	1.90E-02	Gnrh1, Lct
	GO:0016042	Lipid catabolic process	2	1.90E-02	Acaa2, Ces3
	GO:0080134	Regulation of response to stress	3	2.22E-02	Eif2ak1, Fbln5, Tspan8
	GO:0051186	Cofactor metabolic process	2	2.69E-02	Eif2ak1, Acaa2
	GO:0080135	Regulation of cellular response to stress	2	3.49E-02	Eif2ak1, Fbln5
	GO:0071363	Cellular response to growth factor stimulus	2	3.94E-02	Ramp2, Fgfr4
	GO:0048583	Regulation of response to stimulus	5	4.70E-02	Ramp2, Flbn5, Eif2ak1, Fgfr4, Tspan8
	GO:0070848	Response to growth factor	2	4.81E-02	Ramp2, Fgfr4
Molecular function	GO:0016836	Hydro-lyase activity	2	0.000481	Aco2, Alad
	GO:0016835	Carbon-oxygen lyase activity	2	0.00106	Aco2, Alad
	GO:0016829	Lyase activity	2	0.0102	Aco2, Alad
	GO:0020037	Heme binding	2	0.0153	Eif2ak1, Cyb5
	GO:0046906	Tetrapyrrole binding	2	0.0164	Eif2ak1, Cyb5
	GO:0044822	Poly(A) RNA binding	4	0.0255	Serpinh1, S100a4, Acaa2, Slc25a11
	GO:0003723	RNA binding	4	0.0313	Serpinh1, S100a4, Acaa2, Slc25a11
Cellular component	GO:0005788	Endoplasmic reticulum lumen	2	2.38E-03	Ces3, serpineh 1
	GO:0044432	Endoplasmic reticulum part	3	3.15E-02	Ces3, serpineh 1, Cyb5
	GO:0005739	Mitochondrion	4	3.94E-02	Aco2, Mdh1, Slc25a11, Acaa2

GO_id with a p value of <0.05 was selected.

**Table 7:** Signaling pathways predicted to be regulated by altered mRNAs in rat ARDS

**Term**	**Number of genes**	**p-value**
Glyoxylate and dicarboxylate metabolism	2	0.000868
Galactose metabolism	2	0.00172
Citrate cycle (TCA cycle)	2	0.00327
Pyruvate metabolism	2	0.00475
Metabolic pathways	8	0.00753

Analysis of KEGG pathway enrichment in the altered mRNAs in rat ARDS was performed by STRING analysis. Pathways with a p value of < 0.05 were selected.

### Correlation of expression profiles between miRNAs and mRNAs

**Figure 5:**
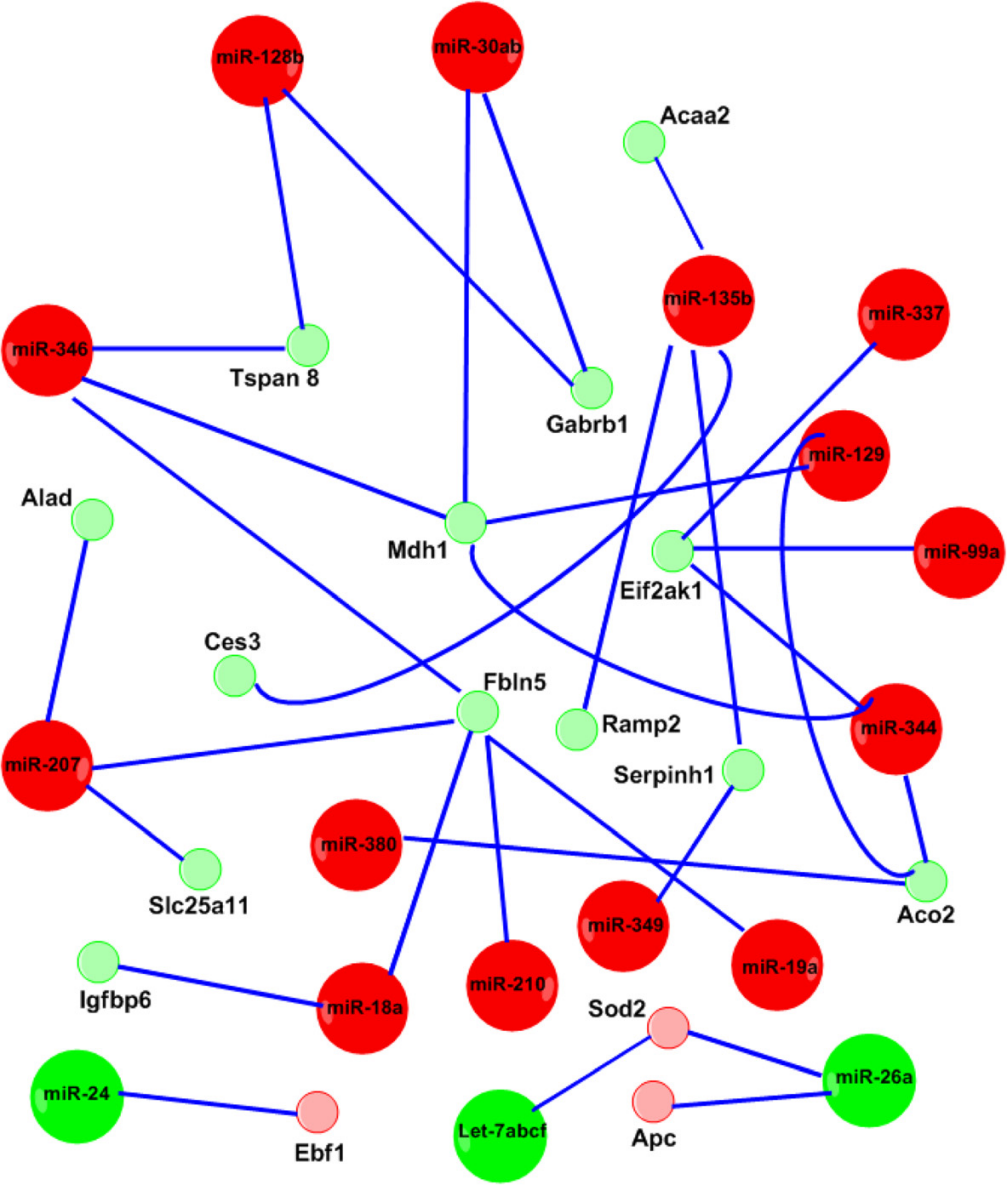
**Interaction network of miRNAs and mRNAs in ARDS.** The miRNA-mRNA interacting network was constructed using the altered mRNAs and miRNAs in ARDS identified in our microarray analyses. The mRNAs were the predicted targets of miRNAs and inversely correlated with miRNAs. Red: up-regulated miRNAs; Green: down-regulated miRNAs; Pink: up-regulated mRNAs; Light green: down-regulated mRNAs.

**Figure 6:**
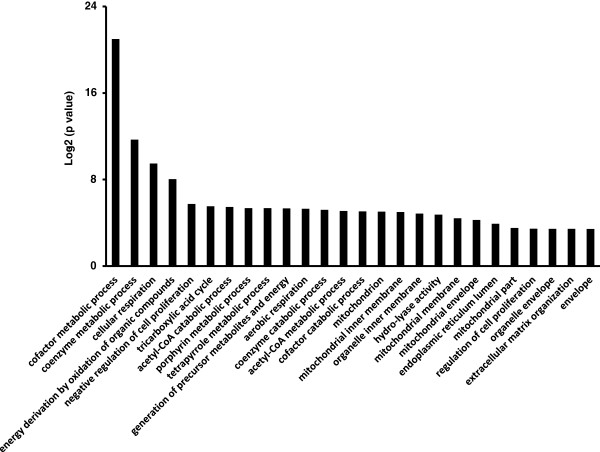
**Identification of functional categories of mRNAs targeted by altered miRNAs in rat ARDS.** GO analysis was performed on mRNAs which were identified by Targetscan or miRanda, and inversely correlated with miRNAs. Only categories with a p-value less than 0.05 were included. The negative log2 of the p-value was plotted on the Y-axis.

Systematic analysis on the interactions of miRNA and mRNA using microarray data could give us information on the role of miRNAs in ARDS. Having performed miRNA and mRNA microarray profiling on the same samples, we were able to analyze the correlation between the identified altered miRNAs and mRNAs. We first predicted the genes targeted by the altered miRNA in ARDS using Targetscan (http://www.targetscan.org) and miRanda (http://www.microrna.org). Then, we compared the predicted miRNA targets with the differentially expressed mRNAs. Table 8 listed the miRNA-mRNA pairs with the inverse correlation of up-regulated miRNAs and down-regulated mRNAs as well as the down-regulated miRNAs and up-regulated mRNAs. Figure 5 showed the graphic presentation of the pairs. miRanda predicted more targets than Targetscan. The predicted miRNA binding sites in the target mRNAs by both programs were in the same location. However, miRanda predicted two rno-miR-128b binding sites and TargetScan only predicted one in the 3’-UTR of Gabrb1. Among the 11 up-regulated mRNAs, Ebf1 (Early B-cell factor 1) was inversely correlated to miR-24. Apc and Sod2 were inversely correlated with miR-26a. Sod2 was inversely correlated with Let-7a, b, c, f. We also identified the inverse correlation of many up-regulated miRNAs and down-regulated mRNAs. miR-346, miR-135b, miR-30a/b, miR-344, and miR-18a had more than one mRNA target. Gabrb1 (Gamma-aminobutyric acid (GABA-A) receptor, subunit beta 1), Sod2, Eif2ak1 (Eukaryotic translation initiation factor 2-alpha kinase 1), Fbln5 (Fibulin 5), and Tspan8 were targeted by multiple miRNAs.GO analysis was applied to the up- and down-regulated genes that were inversely related to the altered miRNAs. We found that cofactor and coenzyme metabolic processes were the top GO categories of these mRNAs (Figure 6).

**Table 8:** Inverse correlation of mRNAs and miRNAs

**miRNA**		**Targetscan**	**miRanda**	**mRNA**
**Up-regulated**	miR-346	Tspan8	Tspan8	**Down-regulated**
		Mdh1	Mdh1	
			Fbln5	
	miR-135b		Acaa2	
			Ces3	
			Ramp2	
			Serpinh1	
	miR-99a		Eif2ak1	
	miR-210		Fbln5	
	miR-19a		Fbln5	
	miR-30ab	Gabrb1	Gabrb1	
			Mdh1	
	miR-128b	Gabrb1	Gabrb1	
		Tspan8	Tspan8	
	miR-207	Alad	Fbln5	
		Slc25a11		
	miR-344		Aco2	
			Mdh1	
			Eif2ak1	
	miR-380		Aco2	
	miR-337		Eif2ak1	
	miR-18a		Fbln5	
			Igfbp6	
	miR-349	Serpinh1		
	miR-129		Aco2	
			Mdh1	
**Down-regulated**	miR-24	Ebf1		**Up-regulated**
	miR-26a	Apc	Sod2	
	Let-7abcf		Sod2	

Tspan8: Tetraspanin 8; Mdh1: Malate dehydrogenase; Fbln5: Fibulin 5; Acaa2: Acetyl-Coenzyme A acyltransferase 2; Ces3: Carboxylesterase 3; Ramp2: Receptor activity modifying protein 2; Serpinh1: Serine (or cysteine) proteinase inhibitor, clade H, member 1; Eif2ak1: Eukaryotic translation initiation factor 2-alpha kinase 1; Gabrb1: Gamma-aminobutyric acid (GABA-A) receptor, subunit beta 1; Alad: Aminolevulinate, delta-, dehydratase; Slc25a11: Solute carrier family 25 (mitochondrial carrier; oxoglutarate carrier), Member 11; Aco2: Aconitase 2; Igfbp6: Insulin-like growth factor binding protein 6; Ebf1: Early B-cell factor 1; Apc: Adenomatosis polyposis coli; Sod2: Superoxide dismutase 2.

### Mapping miRNAs to signaling pathways

DIANA-mirPath is a web-based computational tool to identify signaling pathways regulated by miRNAs [32]. The software compares each set of miRNA targets with all known KEGG pathways to identify the number of miRNA target genes in the pathways. ErbB, MAPK, and WNT signaling pathways had high scores and were likely to be controlled by the altered miRNAs in ARDS (Table 9).

**Table 9:** Signaling pathways predicted to be regulated by altered miRNAs in ARDS

**Pathway**	**Target number**	**Score**
Axon guidance	57	31.92
ErbB signaling pathway	37	19.04
MAPK signaling pathway	80	17.12
Focal adhesion	64	16.73
Regulation of actin cytoskeleton	67	15.37
Colorectal cancer	34	14.74
Chronic myeloid leukemia	31	14.18
Wnt signaling pathway	48	12.55
Glycan structures - biosynthesis 1	40	12.39
Glioma	26	12.37
Pancreatic cancer	28	11.05
Renal cell carcinoma	26	9.49
TGF-beta signaling pathway	31	9.33
Small cell lung cancer	30	8.79
Long-term potentiation	24	8.68
Oxidative phosphorylation	6	8.34
Prostate cancer	30	8.22
Circadian rhythm	8	8.17
Arachidonic acid metabolism	1	7.92
mTOR signaling pathway	20	7.78
Antigen processing and presentation	3	7.3
Adherens junction	25	7.01
Acute myeloid leukemia	21	6.96
T cell receptor signaling pathway	29	6.00
Melanoma	23	5.72
Tryptophan metabolism	1	5.68
Endometrial cancer	18	5.31
Glycosphingolipid biosynthesis - neo-lactoseries	8	5.01
Type II diabetes mellitus	16	4.82
Complement and coagulation cascades	4	4.78
GnRH signaling pathway	28	4.76
Insulin signaling pathway	37	4.44
Melanogenesis	28	4.40
Non-small cell lung cancer	17	4.25

Mapping all the changed miRNAs in ARDS to signaling pathways was performed by DIANA-mirPath software (http://diana.cslab.ece.ntua.gr/pathways). p < 0.01 was the statistical cutoff for DIANA-miRpath analysis. Target number: The number of miRNA target genes in a given pathway. Score: Enrichment statistical score, the negative natural logarithm of the P-value (-ln P).

## Discussion

ARDS is a respiratory disease linked to numerous factors including cytokines, oxidants, and growth factors [33-37]. Functional genomics approaches provide novel insights into understanding gene-environmental interactions controlling this complex process. In our present study, we aimed to identify genes that play critical roles in regulating the pathogenesis of ARDS, and to determine how miRNAs contribute to the regulation of these genes. Key to our approach was microarray analyses to obtain mRNA and miRNA expression profiles in ARDS.

The expression profiles of both miRNAs and mRNAs allow us to determine whether there is a correlation between the expression levels of miRNAs and target mRNAs. We found that up-regulated miRNAs (miR-346, miR-135b, miR-30ab, miR-344, miR-18a, miR-99a, miR-210, miR-207, miR-18a, and miR-129) in ARDS were inversely correlated with the expression of their predicted targets such as Gabrb1, Mdh1, Eif2ak1, Fbln5, and Tspan8. miR-346, miR-135b, miR-30ab, miR-344, and miR-18a were inversely correlated with more than one mRNA targets. Gabrb1, Sod2, Eif2ak1, Fbln5, Tspan8 were targeted by several miRNAs. Moreover, we found that the down-regulated miRNAs, miR-26a, miR-24, and miR-Let-7abcf family, were inversely related to their predicted mRNA targets, Sod2, and Ebf1. miRNA expression patterns have previously been investigated in lung injury models. Let-7 is altered in a mouse model of ventilator-induced lung injury [15]. We also found that Let-7 family was down-regulated in ARDS. miR-126, a regulator of angiogenic signaling and vascular integrity, has been reported to be involved in ARDS/ALI and VEGF is identified as a target of miR-126 [13,14]. Moreover, miR-126 also plays a role in neoangiogenesis of adult tissues in response to injury [38]. In the present study, we found that miR-126 was down-regulated in ARDS. However, we did not find the correlation of miR-126 to the identified altered mRNAs in ARDS.

The major aims of the present study were to identify altered miRNAs and mRNAs in rat ARDS through microarray analyses, and to correlate the identified altered miRNAs and mRNAs by computational prediction. One limitation of the current study was that we did not further validate the predicted miRNA-mRNA interactions. However, some of the predicted miRNA-mRNA interactions from the present study can be found in the Tarbase/mirRecords database, which documented experimentally verified miRNA-mRNA pairs. For example, miR-26a-APC pair was experimentally validated [39]. The second limitation was that we did not answer whether these interactions were biologically important *in vivo*. Since miRNA can inhibit the protein translation without mRNA degradation, the third limitation is that our current approach can not identify these interactions between miRNAs and proteins.

Gene ontology and functional annotation analyses facilitate interpreting the biological relevance of mRNA expression profile in ARDS. More than 50% of the up-regulated genes in ARDS were involved in cellular homeostasis. Cells are essentially factories which strictly maintain their intracellular environment so that conditions remain optimal for performing tasks that take place inside the cells. Chemical and ion homeostasis are important to the cells. Thus, it is of interest to hypothesize that the alteration of the genes involved in cellular homeostasis contributes to the pathogenesis of ARDS. We also found that more than 33% of the up-regulated genes in ARDS were involved in the regulation of apoptosis. These genes included Apc, Timp1, and Sod2. Apoptosis of epithelial and endothelial cells has been observed in the lung of ARDS patients [40]. Apoptosis mediators are also increased in the BAL (bronchoalveolar lavage) fluid of ARDS patients [41]. A delayed apoptosis of intra-alveolar neutrophils with a concomitant increased apoptosis of alveolar epithelium increases the severity of lung injury [41]. Moreover, miRNAs are also involved in the regulation of apoptosis. Up-regulation of miR-26a promotes apoptosis in rat neonatal cardiomyocytes via the caspase-3 pathway [42] while down-regulation of miR-26a antagonizes apoptosis by targeting MTDH and EZH2 in breast cancer [43]. Let-7 is also a regulator of apoptosis in tumors [44]. Interestingly, the down-regulated miRNAs, miR-26a and Let-7abcf family in our ARDS model were inversely related to the expression of Apc and Sod2 which were involved in regulation of apoptosis.

Some of the identified down-regulated miRNAs in ARDS are involved in pulmonary fibrosis [20,45] and lung cancer [46], while the up-regulated miRNAs play critical roles in lung development [12] and in the pathogenesis of lung cancer [47-50]. Among these miRNAs, Let-7 is involved in the airway inflammation by directly regulating IL-13 expression [51]. miR-126 controls leukocyte infiltration into inflamed lungs by repressing ALCAM expression [52]. In addition, miR-126 is down-regulated in cystic fibrosis that is characterized by chronic airway inflammation. miR-126 controls TLR2/4 inflammatory signaling pathways by modulating TOM1 expression in cystic fibrosis lung [45].

The cofactor and coenzyme metabolic processes were on the top list of GO category of mRNAs targeted by altered miRNAs. The changes in cofactor metabolism have been reported in ARDS. Hypoxanthine is a key cofactor that accumulates during hypoxia, leading to the production of O_2_^.-^ and H_2_O_2_, and is significantly elevated in the plasma from ARDS patients [53]. C5a-dependent chemotactic activity is also increased in BAL fluid of ARDS patients [54]. Von Willebrand factor antigen (vWF:Ag) in the vascular endothelial cells acts as a ristocetin cofactor and is related to the development of ARDS [55,56]. Leptin, a potential cofactor involved in lung fibroproliferative responses is increased in the BAL fluid of ARDS patients. The elevated levels of leptinin BAL fluid are associated with a higher mortality [57]. These findings suggest that miRNAs may play roles in the pathogenesis of ARDS by targeting genes that regulate cofactor and coenzyme metabolism.

Extracellular signals cause the alterations in gene expression and metabolism in cells via signal transduction. Abnormal activation or inhibition of multiple signaling pathways often results in lung diseases. WNT/β-catenin signaling pathway plays an important role in lung injury and repair [58]. We mapped the altered miRNAs to signaling pathways by software DIANA-mirPath. ErbB, MAPK, and WNT signaling pathways were found to be controlled by these altered miRNAs in ARDS. Interestingly, miR-26a was inversely correlated with the expression of Apc. The adenomatous polyposis coli tumor-suppressor protein, APC encoded by Apc gene is a negative regulator of WNT signaling.

## Conclusion

In the present study, using miRNA and mRNA microarray analyses, we systematically examined the expression of mRNAs and miRNAs in ARDS, and correlated their expression. This is the first report to integrate miRNA expression data with mRNA expression data in ARDS. The identified miRNAs and mRNAs may be critical in the pathogenesis of ARDS.

## Methods

### Rat model of ARDS

**Figure 7:**
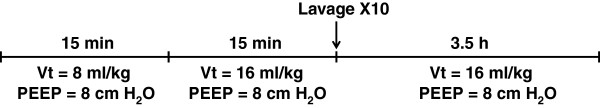
Experimental procedures for a rat model of ARDS.

Adult male Sprague Dawley rats (250–300 gms) were used for this study. All the procedures were approved by Institutional Animal Care and Use Committee at the Oklahoma State University. Rat model of ARDS was induced by saline lavage and mechanical ventilation [25,59] (Figure 7). In brief, rats were anesthetized with intraperitoneal injection of ketamine [80 mg/kg body wight (BW)] and xylazine (10 mg/kg BW). The animals were then placed on a heated water pad maintained at 37°C for the entire length of the procedure. A tracheotomy was performed. A blunt canula was inserted and secured. The animals were ventilated with 100% oxygen at a respiratory rate of 30 breaths/min, a Vt of 8 ml/kg BW, an inspiration: expiration ratio of 1:2, and a PEEP of 3 cm H_2_O. An intramuscular injection of pancuronium bromide (1 ml/kg, BW) was administered for muscle relaxation and preventing spontaneous breathing. After 15 min ventilation, Vt was increased to 16 ml/kg and PEEP to 8 cm H_2_O. The ventilation was continued for an additional 15 min. The lungs were then lavaged with pre-warmed saline (1 ml/30 g BW) for 10 times to deplete lung surfactant and ventilated for additional 3.5 hours. Anesthesia and muscle relaxation were maintained by intraperitoneal administration of ketamine/xylazine, and pancuronium bromide, respectively, at a half of the initial dose every 45 min. At the end of ventilation, the rats were sacrificed by severing the descending aorta. The controls were non-lavaged and non-ventilated rats, which were maintained at room air. To fix the lung, three ml of paraformaldehyde was gently instilled into the left lungs. The instillate sufficiently inflated the lungs. The left bronchus was tied immediately and the left lung en bloc was immersed in the fixative for at least 24 hrs. The procedure resulted in uniform fixation without any artifacts. The right lung was removed for RNA analysis.

### Histopathology

Paraffin-embedded left lung specimens were sectioned, placed on glass slides and stained with hematoxylin and eosin for examination by light microscopy. The histopathological lesions were scored by a board-certified veterinary pathologist in a blinded fashion. The lungs were evaluated for the characteristic histopathological changes of ARDS [25]. In each specimen, alveolar septal necrosis, hyaline membrane formation, intravascular (margination) and intraalveolar (infiltration) accumulation of neutrophils and interstitial (perivascular) and intraalveolar edema were graded according to the distribution and severity of each of the changes. The grades were assigned as follows: 0 = normal; 1 = occasional fields with minimal changes; 2 = occasional fields with changes (mild); 3 = many but not all fields with changes (moderate); 4 = changes in all fields (severe).

### RNA isolation

Small RNAs were isolated from 4 controls and 4 ARDS rat lungs (200 mg) using the mirVana™ microRNA isolation kit (Ambion, Austin, TX) exactly as per the instructions of the manufacturer. Total RNAs were isolated from 200 mg of the same lungs used for small RNA isolation by RNA isolation Kit (Ambion, Austin, TX) exactly as per the instructions of the manufacturer. RNA quality and quantity were assessed with agarose gel electrophoresis, A260/A280 ratio and A260/A230 ratio with spectrophotometer (NanoDrop Technologies, Inc, Rockland, DE). The A260/A280 ratios and A260/A230 ratios for all RNA preparations were greater than 1.9 and 2.0, respectively.

### miRNA microarray

miRNA microarray analyses were performed on an in-house platform developed in our laboratory as previously described [60]. The labeling and hybridization of miRNA were performed with the 3 DNA array 900 miRNA direct kit (Genisphere, Hatfield, PA) according to the manufacturer's protocol. Poly (A) tails were added to the enriched miRNA (150 ng) by poly (A) polymerase. The Fluor 3 or Fluor 5 capture sequences were then ligated to the poly (A)-tailed miRNA. Tagged miRNAs were purified with the MinElute PCR Purification Kit (Qiagen, Valencia, CA). Small RNA samples from control and ARDS lungs were separately tagged with Fluor 3 or Fluor 5 capture sequence. After purification, equal amounts of small RNA from all the samples tagged with the same capture sequence were pooled together as a common reference. To eliminate dye bias, dye-swap was performed. The tagged miRNAs were hybridized to a miRNA microarray slide at 52°C overnight. The array was washed in pre-warmed (52°C) 2 × SSC, 0.2% SDS for 15 min, 2 × SSC for 12 min, and 0.2 × SSC for 12 min at room temperature. After washing, the Alexa Fluor 3 or 5 capture reagents were hybridized to the tagged miRNAs at 62°C for 4 h. The slides were then washed and dried. The hybridized slide was scanned with ScanArray Express (PerkinElmer Life and Analytical Sciences, Boston, MA), and the images were analyzed with GenePix 5.0 pro (Axon Instruments, Inc. Union City, CA). The signal from each spot was normalized to the average signal of the whole block. The highest and lowest signals from the 6 identical probes in the same block were excluded from the data analysis. The geometric average of the remaining 4 signals was considered to be the signal of that particular miRNA. The ratio of sample signal to reference signal was log2 transformed. A quality test was performed with Realspot software developed in our laboratory [61]. The miRNAs with an average quality index of <1 were filtered. The miRNAs that passed the quality test were analyzed with SAM (Significant Analysis of Microarray) to identify miRNAs that were significantly changed in ARDS (q < 0.05) [62]. A fold change of 1.5 rather than 2 was used as a cut-off value in order to identify more miRNAs.

### mRNA microarray

To identify the altered mRNAs in ARDS, we performed mRNA profiling using an in-house printed DNA microarray including 10,000 rat genes [63]. We designed and printed three blocks in each slide so that we can analyze three biological replicates in the same microarray slide. The two-step microarray hybridization was carried out with the 3DNA 50 Expression kit (Genisphere Inc., Hatfield, PA). The hybridized slides were scanned with ScanArray Express. Raw data were extracted from the DNA microarray hybridization images with GenePix Pro 5. Spot image visualization, spot quality evaluation, data normalization, and SAM test for the identification of the differentially expressed genes were performed as previously described using the RealSpot software [61]. The differentially expressed genes between control and ARDS samples were identified based on both fluorescence intensities and normalized log2 ratios. Low quality spots with a mean quality index of less than 1.0 were filtered. The genes that passed the quality test were statistically analyzed by SAM test. The genes with a q value of < 0.05 and a fold change of ≥2 were considered to be the differentially expressed genes.

### miRNA quantitative real-time PCR

Quantitative real-time PCR (qRT-PCR) was used to verify the expression changes of miRNA in ARDS using SYBR Green I [64]. The primers were listed in Table 10. Total RNAs were treated with DNase and purified by Phenol/chloroform extraction and ethanol precipitation. The treated RNAs (2 μg) were poly A-tailed and purified by Phenol/chloroform extraction and ethanol precipitation. Poly A-tailed RNAs were reverse-transcribed into cDNA with polyT adapter as the primer. The thermal conditions for real-time PCR were 95°C for 10 min, followed by 40 cycles of 95°C for 15 sec, 60°C for 30 sec, and 65°C for 30 sec. Data were analyzed using relative quantification based on the comparative C_T_ method. U6 RNA was used as the endogenous reference.

**Table 10:** The primers used for miRNA qPCR

rno-miR-30b-FW	TGTAAACATCCTACACTCAGCTA
rno-miR-99a-FW	AACCCGTAGATCCGATCTTGTG
rno-miR-126-FW	TCGTACCGTGAGTAATAATGCGA
rno-miR-26a-FW	TTCAAGTAATCCAGGATAGGCTA
U6 RNA-FW	GCAAGGATGACACGCAAATTC
General-RE	GCGAGCACAGAATTAATACGAC
PolyT adapter	GCGAGCACAGAATTAATACGACTCACTATAGGTTTTTTTTTTTTVN

### mRNA quantitative real-time PCR

qRT-PCR was used to verify the expression change of mRNAs in ARDS. The primers are listed in Table 11. Total RNA (1 μg) was reverse-transcribed into cDNA with dT17, random hexamer primer, and MMLV reverse transcriptase. Real-time PCR was run in duplicate at 95°C for 10 min, followed by 40 cycles of 95°C for 15 sec, 60°C for 30 sec, and 65°C for 30 sec. The relative expression of genes was determined using the comparative C_T_ method and 18S RNA as a reference.

**Table 11:** The primers used for mRNA qPCR

rTIMP1-up	CAGCAAAAGGCCTTCGTAAAGA
rTIMP1-down	GATCTGATCTGTCCACAAGCAATG
rSOD2-up	GCCTGCACTGAAGTTCAATGG
rSOD2-down	CCCAAAGTCACGCTTGATAGC
rTSPAN8-down	GCAGTTGGGTCCATCATCATG
rTSPAN8-up	GGCTACTTGCAGAAGCAGAATCA
rACAA2-down	ACGTGAGTGGAGGTGCCATAG
rACAA2-up	AAGCTGATCCCACTGCGTATTT
rMDH1-down	CTACTGAAAGCCAACGTGAAGATC
rMDH1-up	AGGCCGTCAGGCAGTTTGTAT
rRAMP2-down	TCATCCTACTGAGGACAGCCTTCT
rRAMP2-up	CAGTTGCACCAGTCCTTGACA

### Bioinformatics analysis

Functional annotation of mRNA expression profile was conducted by DAVID (The Database for Annotation, Visualization and Integrated Discovery) (http://david.abcc.ncifcrf.gov). DAVID provides a tool for annotating biological meaning for input genes. KEGG pathway enrichment in the altered mRNAs was performed by STIRNG analysis (http://string-db.org/). The interactions of the proteins encoded by altered mRNAs were also determined by STRING. STRING is a web-based tool to investigate protein-protein interactions, KEGG pathway, and GO annotation. Targetscan (http://www.targetscan.org) and miRanda (http://www.microrna.org) were used to predict the mRNAs targeted by the altered miRNAs in ARDS. TargetScan predicts mRNA targets of miRNAs based on conserved and unconserved 8 mer and 7 mer sites in the seed region of 3’-UTR of mRNA. miRanda predicts the miRNA binding sites on mRNAs based on a regression model which uses sequence and contextual features of the predicted miRNA-mRNA pair. All the changed miRNAs were mapped to signaling pathways by DIANA-mirPath software (http://diana.cslab.ece.ntua.gr/pathways). DIANA-mirPath utilizes miRNA targets that are predicted with high accuracy and/or experimentally verified targets from TarBase, and perform hierarchical clustering of miRNAs and pathways based on their interaction levels.

## Competing interests

The authors declare that they have no competing interests.

## Authors’ contributions

CH, XX and NRC carried out experiments. CH, XX, NRC, MB and YW analyzed data. CH and NRC drafted the manuscript. LL conceived of the study, participated in its design and coordination and helped to draft the manuscript. All authors read and approved the final manuscript.

## Pre-publication history

The pre-publication history for this paper can be accessed here:

http://www.biomedcentral.com/1755-8794/7/46/prepub

## Supplementary Material

Additional file 1 Terms for up regulated gene. Click here for file
